# Building a human lung from pluripotent stem cells to model respiratory viral infections

**DOI:** 10.1186/s12931-024-02912-0

**Published:** 2024-07-15

**Authors:** Declan L. Turner, Sahel Amoozadeh, Hannah Baric, Ed Stanley, Rhiannon B. Werder

**Affiliations:** 1 Murdoch Children’s Research Institute, Melbourne, 3056 Australia; 2 Department of Paediatrics, University of Melbourne, Melbourne, 3056 Australia; 3 Novo Nordisk Foundation Centre for Stem Cell Medicine, reNEW Melbourne, Melbourne, 3056 Australia

**Keywords:** Respiratory infections, Disease modelling, Stem cell models, Induced pluripotent stem cells, Lung models, Epithelium

## Abstract

To protect against the constant threat of inhaled pathogens, the lung is equipped with cellular defenders. In coordination with resident and recruited immune cells, this defence is initiated by the airway and alveolar epithelium following their infection with respiratory viruses. Further support for viral clearance and infection resolution is provided by adjacent endothelial and stromal cells. However, even with these defence mechanisms, respiratory viral infections are a significant global health concern, causing substantial morbidity, socioeconomic losses, and mortality, underlining the need to develop effective vaccines and antiviral medications. In turn, the identification of new treatment options for respiratory infections is critically dependent on the availability of tractable in vitro experimental models that faithfully recapitulate key aspects of lung physiology. For such models to be informative, it is important these models incorporate human-derived, physiologically relevant versions of all cell types that normally form part of the lungs anti-viral response. This review proposes a guideline using human induced pluripotent stem cells (iPSCs) to create all the disease-relevant cell types. iPSCs can be differentiated into lung epithelium, innate immune cells, endothelial cells, and fibroblasts at a large scale, recapitulating in vivo functions and providing genetic tractability. We advocate for building comprehensive iPSC-derived in vitro models of both proximal and distal lung regions to better understand and model respiratory infections, including interactions with chronic lung diseases.

## Introduction

Respiratory viral infections are the most common cause of human illness, contributing substantially to morbidity, socioeconomic losses, and mortality worldwide [1]. Infections with respiratory syncytial virus (RSV) or influenza virus are leading causes of mortality in children and the elderly [2, 3]. Severe respiratory viral infections in infancy can predispose to later development of chronic lung diseases, including asthma [4, 5], and infections are a common trigger of chronic lung disease exacerbations [6–8]. Respiratory viruses infect individuals of all age groups; however, some respiratory viruses have seasonal oscillation of their outbreaks, with cases of RSV, influenza virus, and coronavirus (CoV) infections peaking during the winter, while rhinovirus, human metapneumovirus, and adenovirus are detected year round [9]. Furthermore, the emergence of novel viral strains, such as severe acute respiratory syndrome (SARS)-CoV-1 and SARS-CoV-2, is a reminder of the potential for new viruses to significantly impact public health.

The primary function of the lungs, to provide oxygen to the blood and expel carbon dioxide, renders the lungs constantly susceptible to inhaled pathogens. Air enters the lungs via the proximal conducting airway composed of the trachea, bronchi, and bronchioles, where it is delivered to the distal gas exchanging zones of the respiratory bronchioles and alveoli. To protect the lung, epithelial cells, resident and recruited immune cells, endothelial and mesenchymal cells coordinate defence against invading pathogens (summarised in Table 1).

**Table 1:** Summary of lung cell type functions at homeostasis and during respiratory viral infections, with reference to production of each cell type from iPSCs

Cell type	Subtype	Function (homeostasis, *additional functions during viral infection*)	Production from iPSCs
Airway epithelial cells	Multiciliated cell	Beating cilia for mucociliary clearance; tight junctions	[33, 34, 100, 113–115].
	Club cell	Secrete antimicrobials and immune modulators; tight junctions	
	Goblet cell	Produce mucous for mucociliary clearance	
	Basal cell	Facultative progenitors of airway epithelial cells	
	PNEC	Relay environmental information to the brain; secrete neuropeptides in response to stimuli	[298, 299]
	Tuft cell	Immune functions	-
	Ionocyte	Ion transport	[300]
Alveolar epithelial cells	Type 2 alveolar epithelial cell	Surfactant production; facultative progenitors of alveolar epithelial cells; immune modulation	[37]
	Type 1 alveolar epithelial cell	Gas exchange	[90]
Innate immune cells	Macrophage	Surfactant recycling; *immunomodulation (proinflammatory and anti-inflammatory); phagocytosis; antigen presentation*	[135, 158, 159, 161–168]
	Neutrophil	*Production of reactive oxygen species; phagocytosis; degranulation; neutrophil extracellular traps*	[194–198]
	cDC	Balancing inflammation; a*ntigen presentation*	[219, 221–223]
	moDC	*Cytokine production; epithelial repair*	[220]
	pDC	*Type I interferon*	[222]
	NK cell	*Killing infected cells; cytokine production*	[236–242]
Endothelial cells	Pulmonary endothelium	Gas exchange; transport of solutes and fluid in the lung; vascular tone; coagulation; *immune cell trafficking; production of cytokines*,* ROS and NOS; pro-coagulation*	[266–269]
	Lymphatic endothelium	Fluid transport in the lung; *immune cell trafficking*	[311]
Fibroblasts	Myofibroblasts	Contraction	[294, 295]
	Lipofibroblasts	Surfactant lipid production	
	Matrix fibroblasts	Extracellular matrix production	

The airway epithelium is a pseudostratified cell layer lining the air-exposed surfaces of the conducting airways, providing a physical barrier between the sterile internal areas of the body and the environment. Specialised multiciliated cells, goblet cells and club cells provide additional protection by trapping dust, allergens, pollutants, and pathogens in mucous and removing them from the airway by mucociliary clearance. Basal cells are the multipotent stem cell population residing at the base of the epithelium and differentiate into multiciliated cells, club, and goblet cells (reviewed [10]). There are additional rare cell types in the airway including pulmonary neuroendocrine cells, which relay environmental information to the brain [11], tuft cells which have specialised innate immune functions [12], and pulmonary ionocytes [13], which have specialised ion transport functions [14]. Additionally, airway epithelial cells operate as sentinels for respiratory infections as they are the primary target for respiratory viral infection [15, 16] where they function to secrete antimicrobial peptides and pro-inflammatory cytokines, to orchestrate appropriate immune responses [17]. In the alveoli, type 2 alveolar epithelial cells (AT2s) provide similar protection to the type 1 alveolar epithelial cells (AT1s) [18–20]. AT1 cells reside adjacent to endothelial cells of the alveolar capillary plexus to facilitate gas exchange with the blood. AT2 cells produce pulmonary surfactant which reduces surface tension preventing alveolar collapse and are facultative progenitors of the alveolus, akin to basal cells of the airway, which actively divide and differentiate into AT1 cells during development and after infection or injury [10, 19].

Supporting the epithelia of the proximal and distal lung is a stromal layer of extracellular matrix (ECM), secreted, maintained, and remodelled by fibroblasts in response to damage or inflammation. The stroma contributes to the maintenance of structural integrity and provides support to tissue resident cells while also remaining navigable for migratory cells, including resident and infiltrating immune cells. A dense network of blood vessels, comprised primarily of endothelial cells, extends throughout the lung. Blood vessels and capillaries are supported by the stroma in the proximal airways and are in direct contact with the basolateral surface of the alveolar epithelium. This facilitates gas exchange and enables the rapid recruitment of innate and adaptive immune cells following infections.

There is a dearth of vaccines or effective antiviral medications for most respiratory viruses. This scarcity of treatments is partly attributable to the absence of tractable human-relevant models for studying viral replication or for identifying host response pathways that could be targeted with pharmaceuticals. Moreover, the need for development of appropriate models is heightened by the human-specific nature of many viruses, diminishing the utility of animal models [21–23]. Likewise, while transformed human cell lines are easy to culture and manipulate, they lack the complexity and diversity needed to recapitulate the physiological reactions of the broad range of human cell types involved in viral pathogenesis [24–26].

**Fig. 1:**
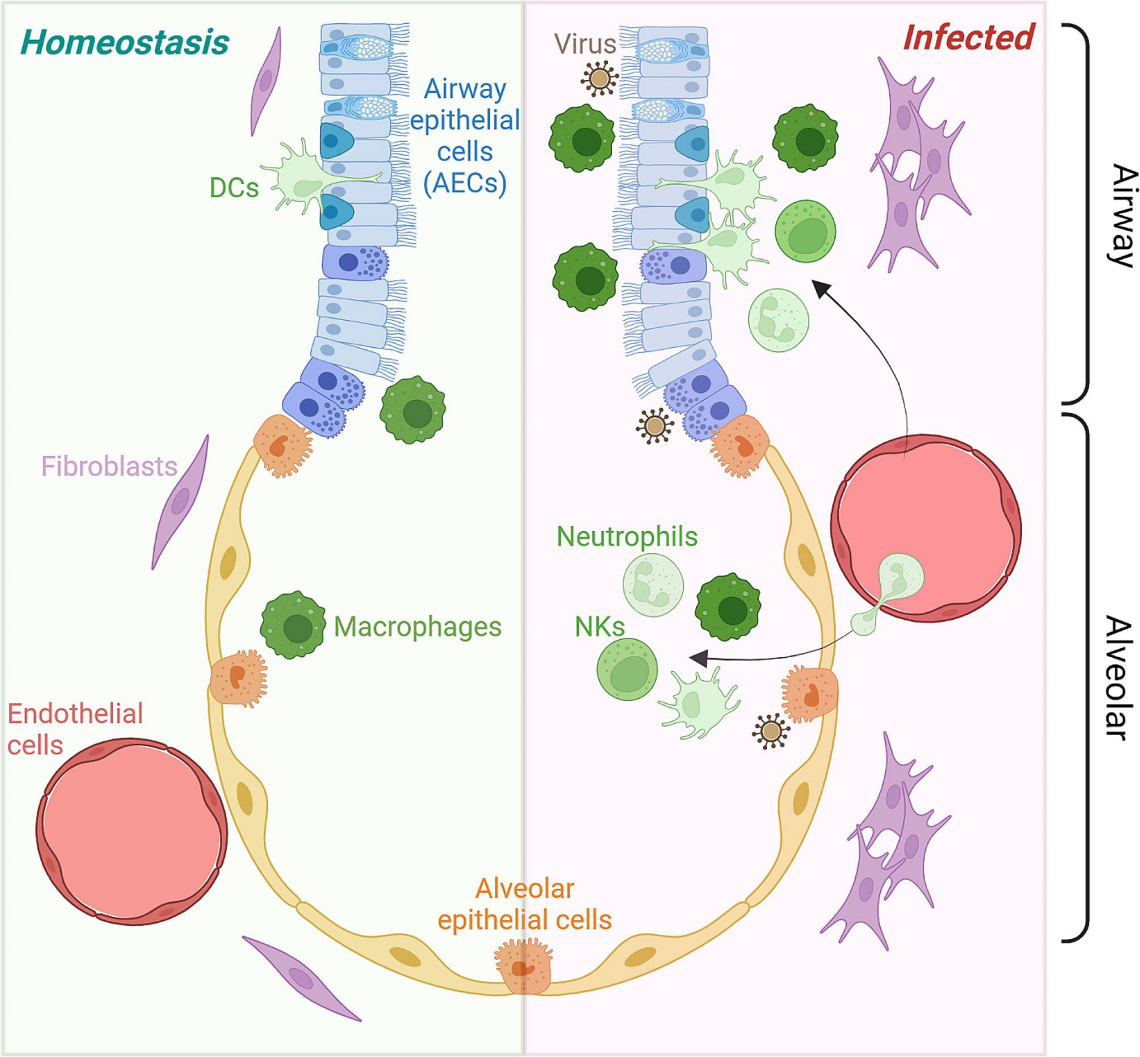
Summary of the cell types and functions of ideal iPSC-derived in vitro airway and alveolar lung models. The airways and alveoli are distinct compartments with unique epithelial cell types and therefore should be modelled separately. We envisage a modular system whereby the airways or alveoli can be modelled at a homeostatic steady state, as well as an inflamed, infected state for comparison. Our ideal system incorporates stromal and endothelial cells, as well as resident (macrophages and dendritic cell [DC]) and infiltrating (neutrophils and natural kill [NK] cell) immune cells. Coupled with the genetic tractability and scalability afforded by iPSC-derived cell types, the intercellular crosstalk, as well as cell intrinsic processes in physiological contexts can be deconstructed with the complete suite of available molecular and genetic tools

Pathogenesis and tissue repair mechanisms following respiratory viral infection are species and cell type specific. To dissect these mechanisms, in vitro models need to recapitulate the cell type diversity, cellular interactions, and the extracellular milieu of both the airway and alveolus in vivo. Primary airway epithelial cells (AECs) have been the predominant in vitro model used to elucidate respiratory viral infection pathogenesis, and have been vital in reliably predicting drug responsiveness, such as cystic fibrosis transmembrane conductance regulator (CFTR) modulators prior to clinical trials [27]. However, challenges in obtaining tissue, limited expansion capability *ex vivo* and poor gene editing potential have restricted their wide-spread application. Furthermore, epithelial-only models do not recapitulate the intricate communication between the epithelium, immune cells, endothelial cells, and stromal cells that ensue following a viral infection and which are central to effective pathogen clearance (Fig. 1). Indeed, the utility of more complex cellular models for understanding respiratory infections has recently been demonstrated using co-cultures of primary airway epithelial cells, pulmonary fibroblasts, endothelial cells and monocytes [28]. Models that mimic the cellular and spatial organisation of the lung will be crucial for discovering and testing new therapeutics, including preventative measures, such as mucosal vaccines.

The discovery of induced pluripotent stem cells (iPSCs) [29] and application of directed differentiation approaches to derive disease-relevant cell types can overcome these limitations. Generated directly from adult somatic cells, iPSCs undergo reprogramming by introducing pluripotency factors [30]. Their self-renewal capacity, ease of access, and potential to differentiate into any desired cell type has enabled the study of lung diseases caused by genetic disorders and/or environmental triggers [31–44]. Of note, iPSCs can be differentiated into lung cell types of interest, including lung airway and alveolar epithelial cells, immune cells, endothelial cells, and fibroblasts, at near limitless scale. Importantly, most of these in vitro derived cell types recapitulate functions of their in vivo counterparts, but with the added benefit of being amenable to genetic manipulation, greatly expanding the accessibility and utility of such models for diverse applications. In addition, iPSC-based platforms would enable all cell types to share a common and consistent genetic background, eliminating variability that is inherent within any system that requires primary material.

The ultimate goal of in vitro lung models is to move beyond traditional observational studies and to gain insights into disease mechanisms by utilising the full suite of molecular and genetic tools available. For example, CRISPR machinery could be deployed in an inducible and cell-type specific manner for high throughput genetic screens to uncover host mechanisms underlying respiratory viral infections [41]. Further, the application of diverse reporters and biosensors offer an enormous diversity of tools to deconstruct mechanisms across space, time, and cell type at unprecedented scale in a physiologically relevant context. In our opinion, combining cell types of interest derived by directed differentiation of iPSCs [45] offers the best path forward for achieving these goals.

In this review, we discuss the development of iPSC-derived lung epithelial cell models and their application to study respiratory viral infections. We also summarise the roles of non-epithelial cell types in respiratory infections as well as their ontogeny in the context of generating iPSC-derived progeny. We draw on this information to propose a roadmap for building a complete iPSC-derived in vitro model of the proximal and distal lung to model existing and emerging respiratory infections, and their interaction with chronic lung diseases.

## Epithelium

Primary airway epithelial cells (AECs) in vitro have been used extensively to interrogate human respiratory infection mechanisms of pathogenesis in a physiologically relevant context [46, 47]. These protocols expand basal cells from brushings, which can be cryopreserved before undergoing terminal differentiation [48]. To recapitulate the pseudostratified airway epithelium, basal cells are matured in air-liquid interface (ALI) cultures or 3D spherical organoids [49, 50]. Airway epithelial ALI cultures have become a mainstay of respiratory research and applied in diverse contexts from drug testing, particle and vapour exposure, toxicity testing and host pathogen interactions (reviewed [49, 51–56])). ALI differentiated cultures are fundamentally distinct from undifferentiated basal cells, illustrated by differential viral growth kinetics and cytokine responses to respiratory virus infection [57, 58]. During infection, respiratory viruses first encounter the apical side of the epithelium, often gaining cellular entry through apical cell surface receptors. This aspect of the viral replication cycle can be recapitulated using ALI cultures, with studies showing that a suite of respiratory viruses preferentially infect via the apical cell surface [59–64]. Notably, AEC ALI cultures confirmed the ciliated cell tropism for parainfluenza virus 3 [60], human metapneumovirus [65], human rhinovirus (HRV) [66, 67], SARS-CoV-1 [62], SARS-CoV-2 [68, 69] and RSV [64, 70]. The cell type specific tropism of respiratory viruses also highlights the essential need to validate therapeutic interventions in appropriate models. For example, hydroxychloroquine showed potent inhibition of SARS-CoV-2 infection of Vero E6 cells [71–73] but limited efficacy in clinical trials of COVID19 patients [74], likely through TMPRSS2 [75, 76], which is highly expressed in the airway epithelium [77, 78], but absent from Vero E6 cells.

Airway epithelial spheroid cultures are a commonly used alternative to the ALI platform. In this system, basal cells are suspended in matrix scaffold where they divide, subsequently forming spheres containing differentiated airway epithelial cells [79]. As for AEC ALIs, airway organoids have also been used to model respiratory infections (reviewed [80, 81]). Matrix-embedded airway organoids develop an epithelium with the apical surface facing the lumen, which complicates infection models since respiratory viruses infect from the apical side. Therefore, organoids must be removed from the matrix, dissociated for infection, and then re-embedded to reassemble their native topology while infected. Direct comparison of 3D airway organoids versus ALI systems confirmed robust infection at ALI and minimal infection in 3D [82], with the ALI system performing equally or better for evaluation of cellular tropism and fitness [83, 84].

Models using primary alveolar epithelial cells are scarce for two major reasons. Firstly, alveolar epithelial cells are not easily accessible [85]. Secondly, AT1 cells are extremely fragile, and most are destroyed during tissue dissociation and processing. Despite these hurdles, a limited number of models have been developed. Isolated primary AT2s differentiate to AT1 cells in culture with the addition of serum [86–89] and recently developed defined conditions [90]. Using this system, tropism, replication and inflammatory responses to SARS-CoV-1 and influenza virus in the alveolar epithelium has been explored [91–94]. Alternatively, AT2s can be maintained in spheroid cultures either in the presence of fibroblast feeder cells [19, 95], or in feeder-free, chemically defined culture conditions [88, 96–99] to study cellular responses to SARS-CoV-2 [88, 96, 98, 99], although it should be noted that these systems also have the same limitations of spherical airway organoid cultures discussed above.

Primary epithelial cells have inherent restraints, chiefly stemming from difficulties related to their acquisition and to their restricted potential for prolonged growth in vitro [100]. This limitation makes it challenging to obtain cells with a consistent genetic background for ongoing studies or to compare results across different laboratories. Additionally, difficulties in generating clonal populations hinder reliable gene editing of primary airway or alveolar epithelial cells in vitro. Moreover, accessing tissue, especially from patients with rare or debilitating conditions, presents an almost insurmountable barrier to mechanistic research. In contrast, lung epithelial cells derived from induced pluripotent stem cells (iPSCs) or human embryonic stem cells overcome these limitations while offering further advantages.

**Fig. 2:**
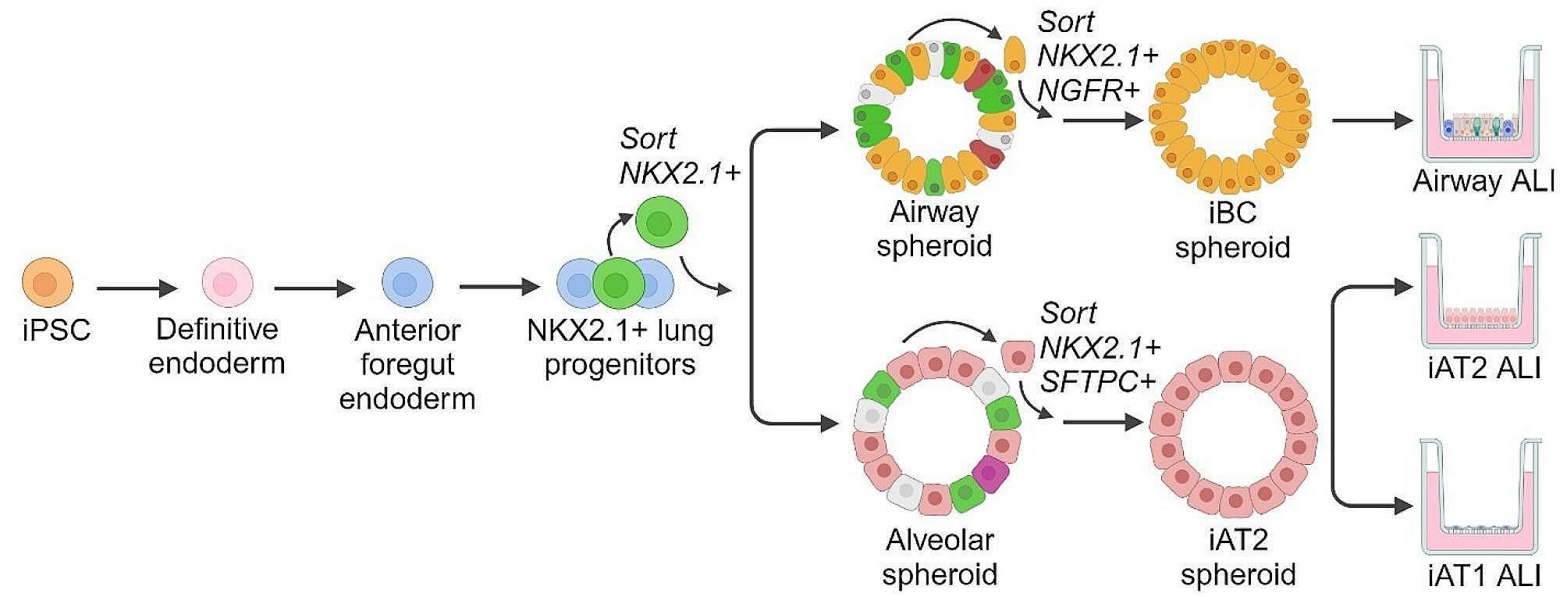
Schematic of directed differentiation of iPSCs to airway and alveolar epithelium. Differentiation of lung epithelial cells from induced pluripotent stem cell (iPSCs) begins with the induction of definitive endoderm and its subsequent specification to anterior foregut endoderm. Expression of the transcription factor NKX2-1 marks the appearance of bipotent lung progenitor cells, capable of forming both airway epithelial and alveolar epithelial cells. To generate airway epithelial cells, lung progenitors are first differentiated to basal cells that, following enrichment by flow cytometry, can be maintained as spheroids for up to ten passages. Plating these basal cells in air-liquid interface (ALI) cultures with specific differentiation media promotes the formation of a mature pseudostratified epithelium. To generate alveolar epithelial cells, lung progenitors are differentiated to type 2 alveolar epithelial (iAT2) cells using specific growth factors and small molecules. Once formed, iAT2s can also be expanded as 3D spheroids for up to 9 months. When required, iAT2s can be seeded at ALI to induce further maturation. In addition, iAT1 cells can also be generated from iAT2s at ALI using specific media and seeding conditions

iPSCs have the capacity to differentiate into any cell type of the body. Directed differentiation of iPSCs in vitro to lung epithelium recapitulates aspects of lung ontogeny, leveraging decades of foundational research on lung development (reviewed [101]). iPSCs are initially specified to definitive endoderm through activin signalling [102, 103] (Fig. 2). Anterior foregut endoderm [104] and primordial lung progenitors expressing NKX2.1 are subsequently derived [42, 105–109]. Enrichment of lung progenitors can be achieved by FACS purification of cells expressing an NKX2.1-fluorescent reporter gene or specific cell surface marker combinations [33, 42]. At this juncture, bipotent lung progenitors can differentiate to airway or alveolar epithelium, depending on the level and duration of WNT signalling (Fig. 2) [34, 109–112]. Airway basal cells can be further purified through cell sorting, serially passaged, and plated as required at ALI, where they differentiate to a pseudostratified airway epithelium containing multiciliated cells, club, and goblet cells [34, 100, 113–115]. Alternatively, WNT activation promotes AT2 cell specification [38, 42, 106, 116–120]. ALI culture of iAT2s induces further maturation by reducing cell cycling and promoting the formation of tight junctions [31, 121]. Recently, iAT1 cells have been derived from iAT2s by manipulating the level of nuclear YAP signalling. iAT1 cells derived by this pathway had a canonical flattened morphology and exhibited increased expression and secretion of alveolar signalling ligands important for mesenchymal and endothelial homeostasis [90].

iPSC-derived airway and alveolar epithelial models have been deployed to study respiratory viruses, including SARS-CoV-2. In ALI cultures, iPSC-derived AECs infected with SARS-CoV-2 mount a robust interferon response. Within these cultures, multiciliated cells are the primary target of infection, mirroring findings from ALI cultures of primary AECs [122]. Similarly, SARS-CoV-2 infection of iAT2 cells in ALI cultures elicits an epithelial intrinsic inflammatory response and loss of the mature alveolar program, demonstrated by downregulation of surfactant protein genes [123]. In these experiments, virions were observed in lamellar bodies, illustrating the potential importance of this AT2-specific organelle in COVID19 pathogenesis. Moreover, analysis of SARS-CoV-2 infected iAT2 cultures revealed dysregulation of cellular organisation and lipid metabolism. Significantly, these perturbations were not detected in commonly used immortal cell lines, A549, Caco-2 and Vero E6 infected with SARS-CoV-2 [35, 124], highlighting the potentially greater physiological relevance of the iPSC-based model systems.

The scalable nature of iPSC-derived lung epithelial cells makes them an ideal platform for drug screening, demonstrated by recent studies that have used these systems to uncover novel inhibitors of SARS-CoV-2 entry or replication [35, 125, 126]. Furthermore, the genetic tractability of iPSCs has underpinned recent investigations into SARS-CoV-2 entry mechanisms in airway or alveolar epithelial cells [127, 128]. Together these studies highlight that iPSC-derived lung epithelial cells not only provide insights into the biology of their in vivo counterparts, but also offer additional benefits such as scalability and ease of genetic manipulation to interrogate respiratory virus pathogenesis.

## Innate immune cells

Viral clearance and infection resolution require a coordinated cellular response. This response is initiated by the infected epithelium and the innate immune cells that are both resident in the lung and rapidly recruited to the site of injury. Achieving a balance between an effective antiviral response versus an exaggerated, damaging immune response is paramount. Below we summarise the importance of various innate immune cells for viral clearance, highlighting any studies focused on the interplay between the human epithelium and immune cells. We also briefly survey iPSC directed differentiation protocols for creating innate immune cells of interest.

### Macrophages

Macrophages are responsible for maintaining lung homeostasis, balancing inflammation, promoting regeneration, and countering infections. They are professional phagocytes and antigen presenting cells, absorbing and digesting pathogens and debris, while also initiating adaptive immune responses by presenting antigens to T lymphocytes in tissues [129, 130]. Macrophages are versatile; capable of both producing proinflammatory and chemotactic signals at sites of respiratory infection, as well as producing anti-inflammatory mediators, and fostering tissue repair in the aftermath of injury [131, 132]. Commonly, macrophages are categorised as having two distinct phenotypes; proinflammatory (M1) and anti-inflammatory (M2) [133], although the full spectrum of phenotypes is not captured by these binary classifications [134]. Nevertheless, irrespective of their designation as M1, M2 or something in between, the diverse range of macrophage effector functions can be traced back to microenvironmental stimuli [135].

Studies in mice demonstrate that at birth, tissue resident macrophages in the lung trace their origins back to progenitor cells in the embryonic yolk sac [136–138]. While extensive studies have demonstrated the self-renewing characteristics of these embryonically-derived macrophages [139–141], it is noteworthy that monocytes originating in the bone marrow play a pivotal role in replenishing the macrophage population in the lung, particularly during periods of infection and inflammation [142–144]. Indeed, in healthy aging, the alveolar macrophages in the human lung are replenished entirely from circulating monocytes [145].

Until recently, models employed for human macrophage studies were mostly confined to immortalized cell lines, and peripheral blood monocyte derived macrophages (MDMs) (reviewed in [135]). While MDMs can be readily obtained in large numbers from human donors, they cannot be maintained in culture for extended periods, and inconsistency is introduced by donor variability, owing to different genetics and infection history [137, 138, 141, 146]. In order to overcome the barriers existing in human macrophage studies, increasing efforts have focused on deriving macrophages from iPSCs [135, 147].

**Fig. 3:**
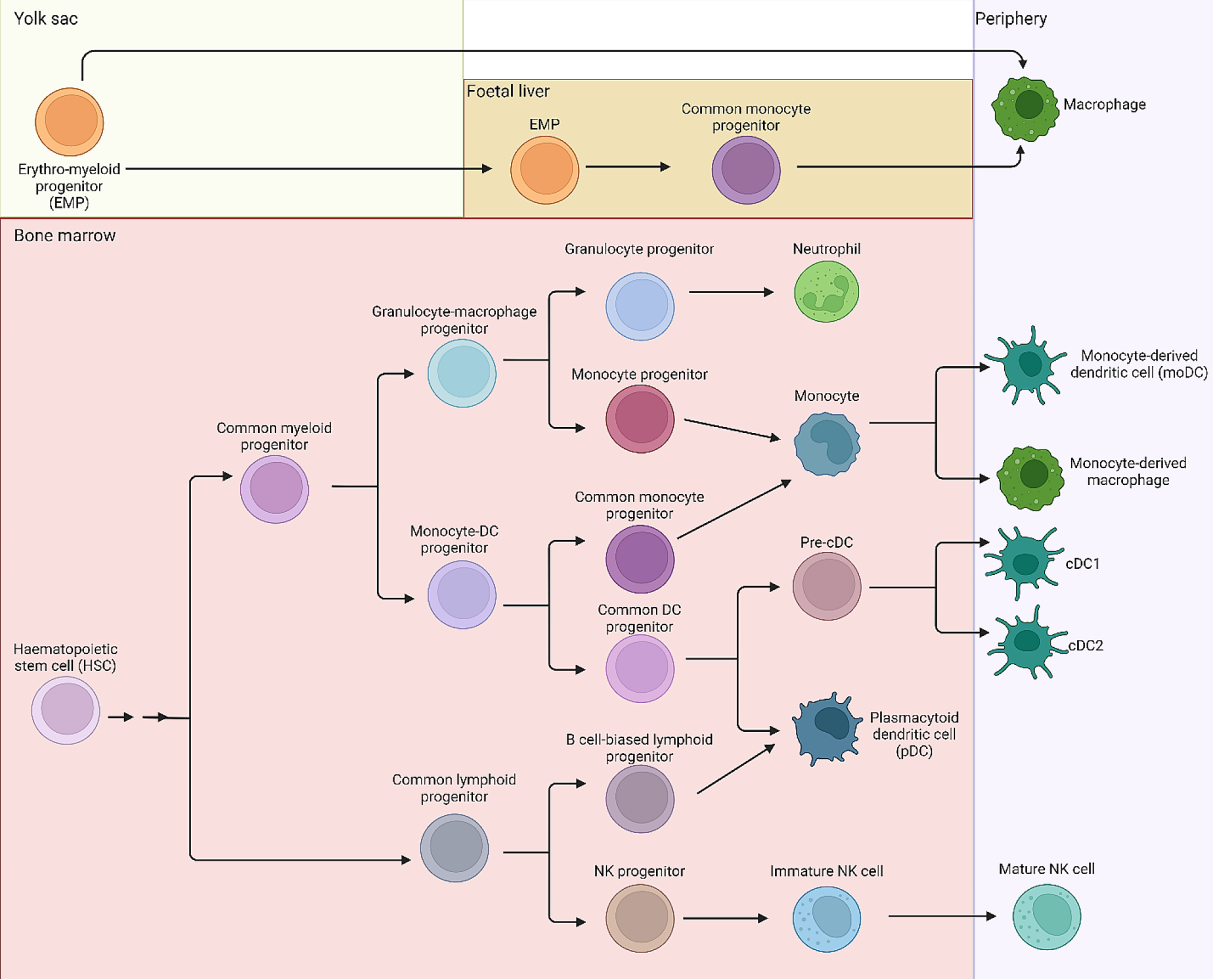
Summary of the haematopoiesis of innate immune cells of interest. Primitive haematopoiesis commences in the yolk sac, giving rise to erythro-myeloid progenitors (EMPs) which can mature into progenitors in the periphery, or through the foetal liver. Definitive haematopoiesis, which includes the production of haematopoietic stem cells (HSCs), can give rise to neutrophils, monocytes (including monocyte-derived dendritic cells [moDCs] and monocyte-derived macrophages), conventional and plasmacytoid dendritic cells (cDC1, cDC2 and pDCs) and natural killer (NK) cells

Given the complex nature of macrophage ontogeny, it is worth considering how distinct developmental pathways contribute to the various macrophage populations that must confront respiratory viral infections in the adult human (Fig. 3). The first wave of human haematopoiesis occurs in the yolk sac [138], predominately yielding primitive erythroid cells and macrophages [148–150], including the resident macrophages in the brain, microglia [137]. The yolk sac is also the site of the second wave of blood cell production, which generates erythromyeloid progenitors (EMPs). EMP derived macrophages seed a number of tissues, including the liver, where they undergo maturation and contribute to the generation of tissue resident macrophages [138, 151]. Definitive haematopoiesis, the third wave, primarily originates in the aorta-gonad-mesonephros region and generates multipotent progenitors, including haematopoietic stem cells (HSCs), capable of giving rise to all the erythroid, myeloid, and lymphoid cell types associated with the adult haematopoietic system. After transiting through the foetal liver [138, 152–154], HSCs migrate to the bone marrow, and at birth they become the source of adult haematopoiesis. During adult life, HSCs underpin the continuous production of cells representing all lineages, including monocytes that can further differentiate into MDMs [155–157]. In summary, macrophages are ultimately produced in all waves of haematopoiesis, with embryonic haematopoiesis giving rise to the first lung-resident macrophages, while definitive haematopoiesis through derivation of HSCs then monocytes will produce macrophages in later stages of life.

Numerous protocols for generating macrophages from iPSC (iMacs) have been described [135]. In general, protocols involve the stepwise differentiation of iPSCs to mesoderm, haemogenic endothelium, hematopoietic progenitors and then, in the presence of Macrophage colony stimulating factor (MCSF or CSF-1), into iMacs. Current iMac differentiation protocols can be broadly viewed as emulating either primitive or definitive haematopoiesis. Frequently, differentiation protocols produce iMacs that closely resemble those formed from primitive haematopoiesis, characterized by MYB-independent myeloid differentiation [158, 159]. These protocols are independent of WNT signalling, yielding haematopoietic progenitors that lack the expression of *HOXA* genes [160]. In addition to MCSF, these protocols often include interleukin (IL)-3 [161–166], a factor which acts to drive proliferation of progenitor populations. Alternatively, haematopoietic progenitors generated through WNT-dependent pathways more closely resemble those that arise from intra-embryonic definitive haematopoiesis [167, 168]. Interestingly, iMacs generated through primitive versus definitive routes in vitro display functional differences, including intrinsic inflammatory responses and phagocytosis [168], suggesting they may respond differently to specific immunological challenges, including respiratory viral infections.

Efforts to establish co-cultures of human iPSC-derived lung epithelial cells with macrophages has increased in recent years. iPSC-derived alveolar-like organoids can be combined with macrophages through microinjection [169] or forced aggregation [170], with the iMacs partially augmenting responses to inflammatory stimuli in vitro [169]. Prior polarisation of iMacs to M1 or M2 phenotypes influences iPSC-derived lung epithelial responses to SARS-CoV-2 infection [171]. Similar co-culture approaches have been trialled by combining primary airway epithelial cells and MDMs in 3D [172] or ALI [173]. Although the application of these models to significantly advance the study of respiratory viruses is currently in the early stages, these studies highlight the potential to apply co-cultured iPSC-derived lung epithelial cells and macrophages at large scales.

### Neutrophils

Neutrophils are the predominant cell type recovered from the airways at the peak of respiratory viral infection [174]. Typically, neutrophils migrate to the lungs via the bloodstream in response to an infection, and attack invading pathogens through production of reactive oxygen species, phagocytosis, degranulation and/or neutrophil extracellular traps [175–185]. While neutrophils dominate the early immune response to respiratory pathogens, these defence mechanisms are often maladaptive, leading to collateral damage to the lungs [176, 181, 182, 186–188]. Moreover, recent evidence shows that neutrophilia in the lungs prior to infection actually predisposes to a more severe infection [189]. Given the clear involvement of neutrophils in the early response to respiratory infections, the incorporation of these cells into iPSC-based infectious disease models is vital.

Neutrophils are derived in vivo from HSCs residing in the bone marrow, through differentiation of common myeloid progenitors, into granulocyte-monocyte progenitors then granulocyte progenitors [190, 191] (Fig. 3). Granulocyte colony-stimulating factor and granulocyte-macrophage colony-stimulating factor then commits these progenitors to a neutrophil fate [192, 193]. Thus, protocols to differentiate iPSCs into neutrophils attempt to recapitulate this sequence of events. This first involves the derivation of CD34 + haematopoietic progenitors from haemogenic endothelium [194–198]. The subsequent differentiation and expansion of progenitors differs across protocols, but generally relies on culture with stem cell factor, FMS-like tyrosine kinase 3 ligand and/or IL-3 or IL-6 [194–198]. Nevertheless, all protocols conclude with high concentrations of granulocyte colony-stimulating factor to induce neutrophil commitment and expansion [194–198]. iPSC-derived neutrophils display typical characteristics of neutrophils, including morphology and behaviour (e.g., production of reactive oxygen species, formation of neutrophil extracellular traps, phagocytosis, and chemotaxis) [194, 197]. While these iPSC-derived neutrophils have been applied for genetic disease modelling in vitro [196] or as a cellular therapy in mouse models in vivo [195, 198], to date they have not been applied to the study of respiratory infections.

### Dendritic cells

Dendritic cells (DCs) reside both within the epithelium and dispersed through the lung interstitium and play a central role in integrating innate and adaptive immunity. In the mouse lung, the roles of various subsets of DCs during respiratory infections have been extensively described, including CD11b + conventional DCs (cDC), CD103 + DCs, monocyte-derived DCs (moDC) and plasmacytoid DCs (pDCs) [199–201]. Similar classification has occurred with respect to human DC populations (CD141 + cDC1, CD1c + cDC2, moDCs and pDCs) [202–205]; however, studies describing the role of these subsets during infections with human-specific respiratory pathogens are limited.

The primary purpose of DCs is to capture and process foreign antigens [206]. Following this, DCs migrate to local mediastinal lymph nodes to present antigens to T cells which in turn initiate antigen-specific cellular and humoral immune responses. Beyond this role, subsets of conventional DCs potentiate inflammation by releasing cytokines and chemokines [199, 207]. In vitro models have demonstrated that peripheral blood mononuclear cell-derived DCs augment cytokine production and epithelial repair [208, 209]. Moreover, pDCs are essential for antiviral responses, producing vast quantities of type I interferons [210–212], and the absence of this cell type impairs responses to respiratory viral infections in mice [213–216].

*In vivo*, DC development initiates from HSCs in the bone marrow. HSCs differentiate to common myeloid progenitors, macrophage-DC progenitors, then common DC progenitors, prior to commitment to either pDCs or pre-cDCs (reviewed in [217] (Fig. 3). Pre-cDCs migrate into the periphery where they differentiate to either cDC1 or cDC2s. pDCs can also arise from common lymphoid progenitors [218] (Fig. 3). Finally, moDCs arise in the periphery from monocytes, which can be derived from monocyte-DC progenitor lineages or granulocyte-macrophage progenitor lineages (see Fig. 3).

Several directed differentiation approaches to generate cDCs and moDCs from iPSCs have been developed [219–222]. To recapitulate development, protocols differentiate iPSCs to haematopoietic progenitors, then use IL-4 and granulocyte-macrophage colony-stimulating factor to specify to cDCs [219]. In some cases, Notch signalling is important for differentiation of DCs [221, 223]. iPSC-derived cDCs display phagocytic, T cell stimulatory and cytokine-producing properties [219, 221, 224]. The generation of pDCs has been less well described; however, the exclusion of IL-4 during the differentiation time course appears to be important [222]. Finally, some applications have successfully derived iPSCs from peripheral blood DCs, in an effort to then increase efficiency of iPSC-derived DC differentiation potential and maturation [224, 225]. The intricate ontogeny of DCs is poorly captured by these directed-differentiation approaches, and further work will be necessary to create precise subsets of DCs to build a lung model that is useful to interrogate respiratory viral infections.

### Natural killer cells

Natural Killer (NK) cells are innate immune cells that play a key role in antitumour and antiviral defence [226]. While NK cells in the human lung parenchyma are mainly CD56^dim^ CD16^+^, indicative of a mature phenotype, few express the tissue residency marker CD69 [227], suggesting the lung is populated by circulating NK cells from the blood, rather than a stable pool of tissue-resident NK cells. During respiratory infections, NK cells constrain viral infection by killing infected cells, either through their direct lysis, and/or through the activation of cell death receptors on target cells (reviewed in [228]). NK cells also shape the immune response through production of cytokines, in particular IFN-γ. Genetic deficiencies that influence NK cell numbers or function are associated with the severity of viral infections in the lung [229]. Furthermore, severe lower respiratory viral infections are associated with low NK cell numbers [230, 231], indicating the important role these cells play in the lung’s response to infection.

In the bone marrow, HSCs give rise to common lymphoid progenitors that can differentiate into NK cells, which undergo further maturation in the bone marrow and/or secondary lymphoid tissues [232–235] (Fig. 3). Thus, to recapitulate this development in vitro, iPSC-derived NK (iNK) cell differentiation protocols first derive haematopoietic progenitors [236–240]. Several approaches have been employed to generate iNK cells from iPSCs, including methods that include the use of stromal cell lines [240, 241] or protocols that rely on the formation of embryoid bodies in defined media conditions [236, 239, 242]. IL-15 is an essential cytokine for NK cell differentiation [233] and is employed in all directed-differentiation protocols reported to date. iNK cells express canonical markers including CD56, NKG2D, NKp44, NKp46, CD16, and Killer Ig-like receptors, and display cytotoxic activity against tumour cells [240, 243]. At present, we are unaware of any studies using iNK cells to enhance understanding of respiratory viral infections. Given the capacity of NK cells to detect and kill virally infected cells, examining the interaction between iNK cells and infected epithelial cells promises to be an exciting area of future research.

### Future iPSC-derived immune cells to consider

As highlighted so far, very few studies have utilised models that have incorporated iPSC-derived immune cells with epithelial cells to interrogate disease mechanisms underlying respiratory infections. Where these models have been tested, work has focused on innate immune cell types which are relatively simple to generate in vitro, such as macrophages. However, for some innate immune lineages, robust iPSC differentiation protocols have not been developed (e.g., basophils). Moreover, it is well recognised that adaptive immune cell types also play important roles in the response to respiratory viral infections. The incorporation of these cell types into an in vitro model of lung infections faces both technical and logistical hurdles. For cells of the adaptive immune system, generation of cells representing these lineages only makes sense when the specificity of their antigen receptors can be pre-determined. Although there has been some success in creating iPSC derived CD8 + Cytotoxic T cells with T cell receptors of known specificity [244], the generation of antigen specific CD4 + T cells or antibody producing B cells is still a major challenge. Ideally, the availability of T cells with T cell receptors that recognise specific viral antigens would enable the construction of complex in vitro cultures that could be used to model respiratory viral infections. The continued improvement of protocols generating various innate and adaptive immune cells from iPSCs will allow future research questions surrounding the involvement of these cells in respiratory viral infections to be answered.

## Endothelium

### Endothelial dysfunction occurs during respiratory infections

The pulmonary endothelium has a critical role in gas exchange, transport of solutes and fluid in the lung, vascular tone, coagulation, and immune cell trafficking [245]. Following a respiratory infection, the endothelium is an active player in coordinating immune responses. For instance, the endothelium is crucial in regulating immune cell entry from the bloodstream into the airspace [246, 247]. Cytokines activate endothelial cells to adopt a leaky, proinflammatory phenotype [248], and endothelial cells are themselves a source of cytokines [249]. Moreover, the activated pulmonary endothelium releases reactive oxygen species and reactive nitrogen species to augment leukocyte chemotaxis (e.g., neutrophils) [250]. Following infections, endothelial cells also adopt a pro-coagulation phenotype to limit damage and restrict infection to the lung [251] (see Table 1).

Endothelial cells play a predominant role in the pathobiology of disease during severe respiratory infections, such as acute respiratory distress syndrome. As these infections progress, the endothelial barrier is disrupted due to cytokines, reactive oxygen species, histamine, or mechanical stretch. Moreover, respiratory pathogens themselves can directly damage endothelial cells [252, 253]. This loss of the barrier and increased permeability causes pulmonary oedema, due to movement of fluid into the interstitial space [245], causing dyspnoea. Given the importance of the pulmonary endothelium in response and repair following respiratory infection, the incorporation of endothelial cells into human lung models of infection is likely to be very important.

### iPSC-derived endothelial cells

Pulmonary endothelial cells exhibit extensive heterogeneity based on anatomical location and functional properties, including barrier properties, proliferation, adhesion receptor expression and leukocyte trafficking [254–259]. Endothelial cells in the lung arise through angiogenesis (growth of existing vessels) and vasculogenesis (new formation of vessels) [260–262]. During embryogenesis, the pulmonary endothelium arises from splanchnic mesoderm [263], while lymphatic endothelial cells arise from paraxial mesoderm [264]. Lineage tracing studies in mice have uncovered further heterogeneity in pulmonary endothelial cell specification, with cardiopulmonary progenitors giving rise to smooth muscle and proximal endothelial cells, but not distal capillary endothelial cells which arise from earlier endothelium [265]. The developmental complexity of endothelial cell specification in the lung is important when considering iPSC-derived endothelial cells to study respiratory viral infections.

There are many approaches for generating endothelial cells from iPSCs, including directed differentiation protocols that allow derivation of arterial or venous endothelial cells [266–269]. In general, a limitation is that iPSC-derived endothelial cells remain limited in their ability to form microvasculature [270], although advances in 3D culture may start to address this [271]. While there are no protocols for creating pulmonary patterned endothelial cells, iPSC-derived endothelial cells seeded into decellularized whole lung scaffolds adopt markers of the endogenous endothelium [272]. These studies suggest the adoption of a pulmonary endothelial fate may require the co-culture of iPSC-derived endothelial cells in a “lung promoting” environment, which may also include airway or alveolar epithelial cells. While iPSC-derived airway epithelium matures in the presence of primary pulmonary microvascular cells [273], the effect of the epithelium on iPSC-derived endothelial cells has not been explored. Notwithstanding these challenges, “generic” iPSC-derived endothelial cells have been successfully applied to understand the pathogenesis of lung diseases, such as pulmonary arterial hypertension in vitro [274–278].

## Stroma

### Fibrotic scarring follows severe respiratory infections

Fibroblasts reside in the lung interstitium at low frequency. In response to injury, fibroblasts are activated, leading to an increase in their numbers. Fibroblast activation, which is thought to promote tissue repair, is typically driven by signals from adjacent injured alveolar epithelial cells. Recent studies in mice have shown that during acute respiratory infection, subsets of fibroblasts adopt interferon-responsive, proinflammatory or pro-repair phenotypes [279]. Similarly, fibroblast-derived ECM proteases remodel the lung microenvironment following infection [279, 280]. While this new role for fibroblasts during acute infection is emerging, considerably more is known about their role during wound repair.

Pulmonary fibrosis can develop following severe or persistent damage to the lung induced by respiratory infections, such as acute respiratory distress syndrome and pneumonia [281, 282]. Studies of patients with severe acute respiratory syndrome (SARS) and Middle East respiratory syndrome, both immediately following infection and 15 years later, noted impaired lung function and radiographic findings consistent with pulmonary fibrosis [283, 284]. A similar scenario is unfolding in the wake of the COVID19 pandemic, with pulmonary fibrosis a common feature of Post-Acute Sequelae of COVID-19, also termed “long COVID” [285–287]. Post-mortem examinations of post-COVID19 lungs have revealed hallmark abnormalities such as alveolar epithelial cell dysfunction, fibroblastic foci, alterations in the extracellular matrix, and immune cell infiltration [287]. While these studies demonstrate the association between pulmonary fibrosis and prior severe respiratory infections, the molecular mechanisms linking these two events has not been established, highlighting the need for further mechanistic studies.

### iPSC-derived stromal cells

Lung fibroblasts can be classified based on spatial localization, or function. For instance, fibroblasts can be broadly characterized as adventitial fibroblasts, residing proximally, and alveolar fibroblasts, residing in the distal region of the lung. These spatially distinct populations display distinct characteristics, such as differential expression of ECM proteins [279, 288, 289]. Alternatively, functional heterogeneity enables lung fibroblasts to be classified as either myofibroblasts, lipofibroblasts or matrix fibroblasts. Myofibroblasts have contractile properties, are important in wound repair, and play pathological roles in pulmonary fibrosis [290]. Lipofibroblasts reside in close proximity to, and provide surfactant lipids to AT2s [291]. Matrix fibroblasts generate the lung ECM [292]. During injury, there is evidence that fibroblasts can convert between lipofibroblast and myofibroblast states, demonstrating the plasticity of fibroblast identities [293].

Methods for the derivation of tissue-specific mesenchyme from iPSCs have lagged behind other lung lineages. A recent study by Alber et al. described the generation of the first mouse iPSC-derived lung mesenchyme that express key transcriptional and functional features of in vivo mesenchyme [294]. Co-culture of this lung mesenchyme with iPSC-derived epithelial cells upregulated gene signatures associated with alveolar and adventitial fibroblasts. Moreover, the authors observed this cross talk was bidirectional with epithelial fate influenced too [294]. Similarly, iPSC-derived (non-tissue specific) mesenchyme supports the development of iPSC-derived alveolar epithelial cells [295]. These early forays into generating iPSC derived lung stromal cells represent an important step to recreating the diversity of lung stromal populations. In this context, further work will be required to understand the nature of human iPSC-derived lung mesenchymal cells, particularly how these relate to specific fibroblast subtypes.

## Applications for iPSC-derived models

In establishing iPSC-derived models of the lung, several practical and technical points need to be considered. Practically, working with iPSCs is time-consuming, expensive and requires tacit knowledge through experience. This reality is likely to place such models beyond the reach of research groups whose work is primarily focused on the biology of lung cells, rather than how these cells are made. Technically, differentiation protocols for many of the cell types required to construct a complex lung model are still a work in progress. Directed differentiation protocols for proximal and distal lung epithelium have matured considerably in recent years; however, the occasional emergence of non-lung endoderm cells can occur [33, 296, 297]. Furthermore, rare cell types such as tuft cells, pulmonary neuroendocrine cells and pulmonary ionocytes have limited representation in iPSC-derived AEC models, although recent successes have occurred in this area [298–300]. For the alveolar compartment, co-culture of iAT1 and iAT2 cells, as well as the introduction of mechanical forces [301], which occur during breathing, represents an opportunity to establish more physiologically relevant models. Finally, directed differentiation protocols for some cell types that might be required to complete a proposed model (e.g., stromal cells), are a headwind in the near term; however, this will improve over time as protocols evolve.

**Fig. 4:**
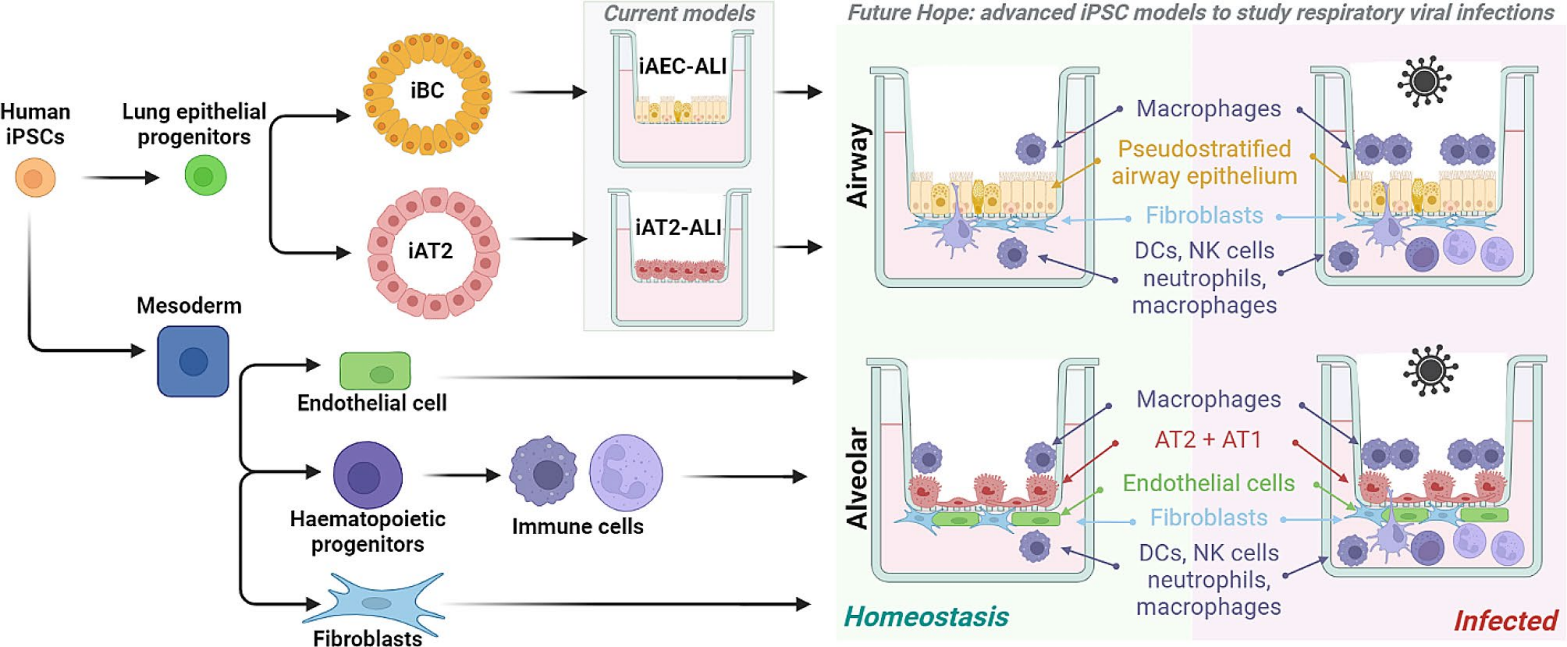
Idealised workflow to create iPSC-derived “mini lungs”. Proposed models will build on existing induced pluripotent stem cell (iPSC) derived airway epithelial cell (iAEC) and type 2 alveolar epithelial cell (iAT2) air-liquid interface (ALI) cultures. Distinct directed differentiation of mesodermal lineages, including endothelial cells, haematopoietic cells, and fibroblasts would be performed, prior to incorporation with epithelial cells to mimic the homeostatic or infected airway or alveolus

In constructing a “mini lung” model (Fig. 4), the optimal combination of immune, stromal, endothelial, and epithelial cells will depend on the research question under consideration, whether that involves specific pathogens or genetic diseases. Likewise, the optimisation of medium composition, extracellular matrix, cell seeding sequence, and cell density will likely be required to enable “mini lung” systems to adequately model different human pathologies. Ideally, advances in cryopreservation methods for each iPSC-derived cell type will allow an “off-the-shelf” approach, freeing researchers from the challenge of coordinating multiple directed differentiation outcomes. We foresee that the technical challenges for establishing iPSC-based platforms will be far outweighed by the applications.

The role of host factors during viral infection are cell type specific, precluding simplistic or one size fits all approaches to characterising virus-host interactions. High-throughput pooled genetic screens are an attractive methodology [302] to probe these complex interactions, enabling cellular genes crucial to the viral replication cycle to be identified in an unbiased fashion. Cognisance of relevant cell types is also important, as it was shown that key genes enabling SARS-CoV-2 infection could vary greatly between cell types [303]. In this context, determining the optimal frequency of different cell types within a complex mini lung model will be a necessary prelude to using these models for drug discovery. Notwithstanding these challenges, iPSC-derived respiratory models will provide an ideal system for physiologically relevant, high-throughput screens for lung diseases (reviewed [304]).

Another application where iPSC-derived lung models show great promise is in modelling genetic diseases, including cystic fibrosis, primary ciliary dyskinesia, alpha-1 antitrypsin deficiency, and interstitial lung disease. Donor-derived iPSCs can be created, and disease-causing mutations corrected in vitro to generate isogenic control lines which are critical for assessing the impact of genetic variants. For instance, pairs of iPSC lines differing only by the presence of a specific disease causing mutation have been generated for cystic fibrosis [34, 305, 306], primary ciliary dyskinesia [34, 307], alpha-1 antitrypsin deficiency [308, 309] and childhood interstitial lung disease [32, 43, 310]. The availability of these paired iPSC lines is critical for mechanistic studies and drug screening protocols, which prior to the advent of iPSC technologies, were challenging because of limitations in obtaining and manipulating primary cells. iPSC-derived models can circumvent these limitations as they enable complex genetic modifications, including the generation of isogenic controls, and provide the scalability required for mechanistic studies and high-throughput screens.

## Conclusions

Respiratory infections inflict an enormous toll in terms of mortality, morbidity, and economic loss, yet comparatively few antiviral drugs, vaccines or therapies exist for these common infections and their associated diseases. The relative dearth of effective therapeutics is due, in part, to the lack of tractable in vitro systems that enable incorporation of relevant human cell types that accurately model the behaviour of their in vivo counterparts. Building models of the lung with iPSC-derived progeny overcomes the limitations of immortalised cells and primary cell systems while retaining the advantages of both, including scalability, ease of gene editing and physiological complexity. Additionally, iPSC-derived models provide an accessible platform for the study of genetic and chronic diseases, and how specific human cell types interact with drugs, toxins, and importantly, respiratory infections. Robust protocols now exist to create iPSC-derived lung epithelial cells which faithfully recapitulate the biology of both the airway and alveolar epithelium. The next step will be to combine these with non-epithelial cell types, such as stromal, endothelial, and immune cells, to create complex “mini lung” models that better reflect the true cellular diversity of the lung. The generation of such complex “mini lung” models has the potential to expand our understanding of how cellular interactions shape the response to viral infections and usher in a new era for respiratory research and therapeutic development.

## Data Availability

No datasets were generated or analysed during the current study.

## Abbreviations

AEC: Airway epithelial cell
ALI: Air–liquid interface
AT1: Type 1 alveolar epithelial cell
AT2: Type 2 alveolar epithelial cell
cDC: Conventional dendritic cell
CFTR: Cystic fibrosis transmembrane conductance regulator
CoV: Coronavirus
DC: Dendritic cell
ECM: Extracellular matrix
EMP: Erythromyeloid progenitor
hESC: Human embryonic stem cell
HRV: Human rhinovirus
HSC: Haematopoietic stem cell
iAT1: iPSC–derived type 1 alveolar epithelial cell
iAT2: iPSC–derived type 2 alveolar epithelial cell
iBC: iPSC–derived basal cell
IL: Interleukin
iMac: iPSC–derived macrophage
iPSC: Induced pluripotent stem cell
M-CSF: Macrophage colony–stimulating factor
MDM: Monocyte–derived macrophage
moDC: Monocyte–derived dendritic cell
NK: Natural killer
pDC: Plasmacytoid dendritic cell
RSV: Respiratory syncytial virus
SARS: Severe acute respiratory syndrome
